# Moonlighting enzymes of *Borrelia burgdorferi*

**DOI:** 10.1128/mbio.00279-25

**Published:** 2025-04-02

**Authors:** Jorge L. Benach

**Affiliations:** 1 Microbiology and Immunology, Stony Brook University Renaissance School of Medicine 12300, Stony Brook, New York, USA; The Ohio State University, Columbus, Ohio, USA

**Keywords:** moonlighting, enzymes, *Borrelia burgdorferi*

## Abstract

Moonlighting enzymes are increasingly recognized in bacteria with dual functions depending on whether they are intracellular or expressed on the surface. Enzymes of the glycolytic pathway are among the most frequently associated with moonlighting functions and lack the signal sequences needed to deliver them to the cell surface. Once these enzymes are on the surface, they perform functions that are associated with pathogenesis and development of infection through interaction with host substrates. One such interaction is adhesion. *Borrelia burgdorferi,* the etiologic agent of Lyme disease, must encounter a wide number of different tissues and substrates from ticks to mammalian hosts to complete its life cycle and persist. The phosphomannose isomerase of this organism has a moonlighting function, interacting with collagen IV, a main component of the basal lamina. It is abundant in the skin, which is the site of the initial infection of *B. burgdorferi*.

## COMMENTARY

Moonlighting enzymes are a group of molecules that perform their canonical functions and can perform other unrelated functions. An important aspect of moonlighting enzymes is that their canonical function is intracellular, whereas the additional function is related to their being expressed on the cell surface. The moonlighting activity is not due to any known molecular alterations, such as splicing, fusions, or proteolytic breakdown. In fact, most of these moonlighting proteins are not different inside the cell or on the cell surface. The field of bacterial moonlighting enzymes has grown markedly since the initial observation that the GAPDH of group A streptococcus had a surface location that permitted attachment to several substrates (1). Many of the bacterial moonlighting enzymes are from glycolysis but also can be chaperones as well as other molecules that have canonical biochemical functions. The field has been the topic of excellent reviews, with the latest trying to understand why these enzymes have been detoured to the cell surface to perform functions unrelated to canonical jobs (2). Most moonlighting enzymes lack the signal sequences that are used to deliver them to the cell surface. It is tempting to speculate that unaltered moonlighting enzymes reach the surface through an undiscovered secretion system. Once on the surface, these moonlighting enzymes can become adhesins for cells or substrates, and this function has been described overwhelmingly for Gram-positive organisms but also for spirochetes and Gram-negative organisms (3–5).

*Borrelia burgdorferi,* the etiologic agent of Lyme disease, must come in contact with a wide number of different tissues and substrates from ticks to mammalian hosts to complete its life cycle and persist. Indeed, the field of *Borrelia* pathogenesis has understood this requirement and has provided substantial evidence for adhesion with excellent reviews (6–9). The genome of *B. burgdorferi* provides the clue for its ability to bind to such a large variety of tissues and substrate molecules. It is among the most A + T rich bacteria with a lysine content of 10.2% and a median calculated isoelectric point of 9.2 (10). Many of the interactions of *B. burgdorferi* with substrates are mediated by cationic lysine residues (11–14), and substrate macromolecules that are known receptors for *B. burgdorferi* have negative electrostatic charges such as plasminogen, integrins, plasma, matrix fibronectin, and extracellular matrix proteins (decorin, collagens, and laminin). Adhesion of this spirochete is probably mediated by electrostatic interactions of cationic ligand proteins and acidic or neutral cell or matrix receptors. To the extent that these electrostatic interactions may have low avidity, this is to the advantage of an organism whose motility is a main defense mechanism.

Recently in *mBio*, a study from Dutta et al. showed that the zinc-dependent enzyme, phosphomannose isomerase (PMI) of *B. burgdorferi*, has a moonlighting function associated with the ability of this organism to cause infection (15). This enzyme catalyzes the interconversion of fructose 6-phosphate and mannose-6-phosphate. This function permits these sugars to be utilized in glycolysis and several other metabolic pathways. As expected, this function is carried out in the cytosol of cells ranging from bacteria to eukaryotes. However, consistent with a common theme for enzymes involved in glycolysis, PMI was also found to be localized in the outer membrane of the spirochete. Localization in the outer membrane was achieved by several approaches, including Triton X-114 phase partitioning, immunofluorescence with antibodies by ELISA and flow cytometry, and proteinase K accessibility assays. Such thoroughness is mandatory for studies of this nature using *B. burgdorferi* as this organism has a frail outer membrane that can lead to ambiguous results. Importantly, PMI also retained its enzymatic activity when localized to the outer membrane. Although *B. burgdorferi* has been shown to adhere to several extracellular matrix components (see above), PMI was found to interact with collagen IV and was of special interest to the pathogenesis of this infection. Collagen IV is found primarily in the basal lamina. Unlike other types of collagens, collagen IV does not form tight helices but rather exists as a sheet in the basement membranes. Importantly, it is particularly abundant in skin and in the basement membrane of the vasculature, and these are anatomical sites preferred by this spirochete (16). These authors used conventional and artificial intelligence-driven structural methods to demonstrate that PMI can dock the sugars of a glycosylated asparagine on collagen IV. This level of structural detail was further enhanced with studies using inhibitors to PMI. Lastly, this study showed that interference with PMI using several approaches can lead to impaired infection.

The interaction between PMI and collagen IV appears to be more specific than those driven by electrostatic charges. The calculated isoelectric point of PMI is between 5.2 and 5.4, and the collagens display roughly similar isoelectric points. PMI is a monomeric and zinc-dependent enzyme, and in its location on the surface of the organism, it becomes a ligand for collagen IV. The structural results suggest an interaction of high affinity, and the substrate is a component of the basal lamina that enhances the importance of these findings.
